# Amino Acid Transporter LAT1 (SLC7A5) Mediates MeHg-Induced Oxidative Stress Defense in the Human Placental Cell Line HTR-8/SVneo

**DOI:** 10.3390/ijms22041707

**Published:** 2021-02-08

**Authors:** Sebastian Granitzer, Raimund Widhalm, Martin Forsthuber, Isabella Ellinger, Gernot Desoye, Markus Hengstschläger, Harald Zeisler, Hans Salzer, Claudia Gundacker

**Affiliations:** 1 Karl-Landsteiner Private University for Health Sciences, A-3500 Krems, Austria; sebastian.granitzer@kl.ac.at (S.G.); raimund.widhalm@kl.ac.at (R.W.); 2 Institute of Medical Genetics, Medical University of Vienna, A-1090 Vienna, Austria; martin.forsthuber@meduniwien.ac.at (M.F.); markus.hengstschlaeger@meduniwien.ac.at (M.H.); 3 Department of Pathophysiology and Allergy Research, Medical University of Vienna, A-1090 Vienna, Austria; isabella.ellinger@meduniwien.ac.at; 4 Department of Obstetrics and Gynecology, Medical University of Graz, A-8036 Graz, Austria; gernot.desoye@medunigraz.at; 5 Department of Obstetrics and Gynecology, Medical University Vienna, A-1090 Vienna, Austria; harald.zeisler@meduniwien.ac.at; 6 Clinical Department of Pediatrics and Adolescent Medicine, University Hospital Tulln, A-3430 Tulln, Austria; Hans.Salzer@tulln.lknoe.at

**Keywords:** LAT1, placenta, HTR-8/SVneo, MeHg, mercury, oxidative stress, GSH

## Abstract

The placental barrier can protect the fetus from contact with harmful substances. The potent neurotoxin methylmercury (MeHg), however, is very efficiently transported across the placenta. Our previous data suggested that L-type amino acid transporter (LAT)1 is involved in placental MeHg uptake, accepting MeHg-L-cysteine conjugates as substrate due to structural similarity to methionine. The aim of the present study was to investigate the antioxidant defense of placental cells to MeHg exposure and the role of LAT1 in this response. When trophoblast-derived HTR-8/SVneo cells were LAT1 depleted by siRNA-mediated knockdown, they accumulated less MeHg. However, they were more susceptible to MeHg-induced toxicity. This was evidenced in decreased cell viability at a usually noncytotoxic concentration of 0.03 µM MeHg (~6 µg/L). Treatment with ≥0.3 µM MeHg increased cytotoxicity, apoptosis rate, and oxidative stress of HTR-8/SVneo cells. These effects were enhanced under LAT1 knockdown. Reduced cell number was seen when MeHg-exposed cells were cultured in medium low in cysteine, a constituent of the tripeptide glutathione (GSH). Because LAT1-deficient HTR-8/SVneo cells have lower GSH levels than control cells (independent of MeHg treatment), we conclude that LAT1 is essential for de novo synthesis of GSH, required to counteract oxidative stress. Genetic predisposition to decreased LAT1 function combined with MeHg exposure could increase the risk of placental damage.

## 1. Introduction

The growing fetus requires amino acids to build up proteins and synthesize non-protein molecules, such as the antioxidative tripeptide glutathione (GSH). The placenta separates the maternal from the fetal circulation and allows the exchange of gases, nutrients, and waste products between mother and child. To ensure adequate fetal supply with the essential biomolecules, the placenta is equipped with a variety of amino acid transporters [1]. However, some of these transporters are known to also mediate the uptake of certain toxicants due to their broad substrate profile [2]. In case of methylmercury (MeHg), heterodimeric amino acid transporters have been implicated into its transport across physiological barriers. These include the L-type amino acid transporter (LAT)1 (subunits LAT1 (SLC7A5) and heavy chain 4F2hc (SLC3A2)), LAT2 (LAT2 (SLC7A8), and 4F2hc (SLC3A2)), and system b (0, +) (b(0, +), AT (SLC7A9), and heavy chain rBAT (SLC3A1)) [3,4,5]. According to these studies, LAT1 seems to be the most important transporter involved in MeHg uptake. The underlying molecular mechanism relies on the structural similarity of MeHg-L-cysteine complexes with the essential amino acid methionine [4,6].

Humans are exposed to MeHg mainly through fish and shellfish consumption. MeHg is the most toxic mercury form for the human body [7,8]. Its cytotoxic properties rely on disruption of the antioxidative system (by reactive oxygen species (ROS) generation and GSH depletion) and induction of apoptosis [9,10,11]. Although MeHg toxicity can affect virtually all organs, its primary target is the developing brain [8,12,13,14].

The human placental barrier is a multilayered structure consisting of the syncytiotrophoblast (STB), which directly contacts maternal blood, the underlying cytotrophoblast (CTB) present as a continuous cell layer in early pregnancy, and the human placental fetal endothelial cells (pFECs) lining fetal blood vessels [15,16].

This placental barrier, however, can protect the fetus from contact with toxins and pathogens only to a limited extent. Unlike other heavy metals, MeHg is very efficiently transported across the placental barrier, suggesting an active transport mechanism [17,18]. Previous findings on the accidental uptake of MeHg (presumably as L-cysteine conjugate) into trophoblast cells by the methionine transporters LAT1 and LAT2 [4,19], as well as the efflux of MeHg conjugated to through ATP-binding cassette transporters, particularly multidrug-resistance protein 1 (MRP1) [20,21], may well explain why MeHg is so efficiently transported from the maternal into the fetal bloodstream.

The effects on placental cells have been scarcely studied. MeHg inhibits the carnitine acetyltransferase activity in human placental STB, and it has antiproliferative, cytotoxic, and apoptotic effects, as determined in various trophoblast cell culture models [4,21,22,23].

In view of the gaps in knowledge, our aim was to investigate the antioxidant defense of placental cells HTR-8/SVneo to MeHg exposure and the role of LAT1 in this response. We investigated whether LAT1 depletion would reduce cellular MeHg uptake and studied the accompanying effects on cell number, cell viability, cytotoxicity, and apoptosis rate. We further explored if alterations in intracellular Hg levels would influence the GSH status that correlates to the potential of placental cells to respond to oxidative stress. It is still unclear whether an extracellular supply of relevant amino acids would promote or inhibit MeHg uptake in placental cells and consequently impact on MeHg accumulation and redox status of placental cells. Thus, we additionally exposed HTR-8/SVneo cells to MeHg in culture medium containing different amounts of cysteine and methionine. We hypothesized that MeHg uptake into HTR-8/SVneo cells could be reduced by (1) removing cysteine from the medium, thereby preventing the formation of the MeHg-L-cysteine complex, or (2) increasing the concentration of methionine, which would compete with MeHg-L-cysteine as LAT1 substrate. As both cysteine and methionine are involved in GSH synthesis [24], we also investigated if the cellular GSH status was dependent on the extracellular availability of these two amino acids.

## 2. Results

### 2.1. LAT1 Protein Expression in the Placenta and Verification of siRNA Mediated Gene Knockdown of LAT1 in HTR-8/SVneo Cells

**Figure 1:**
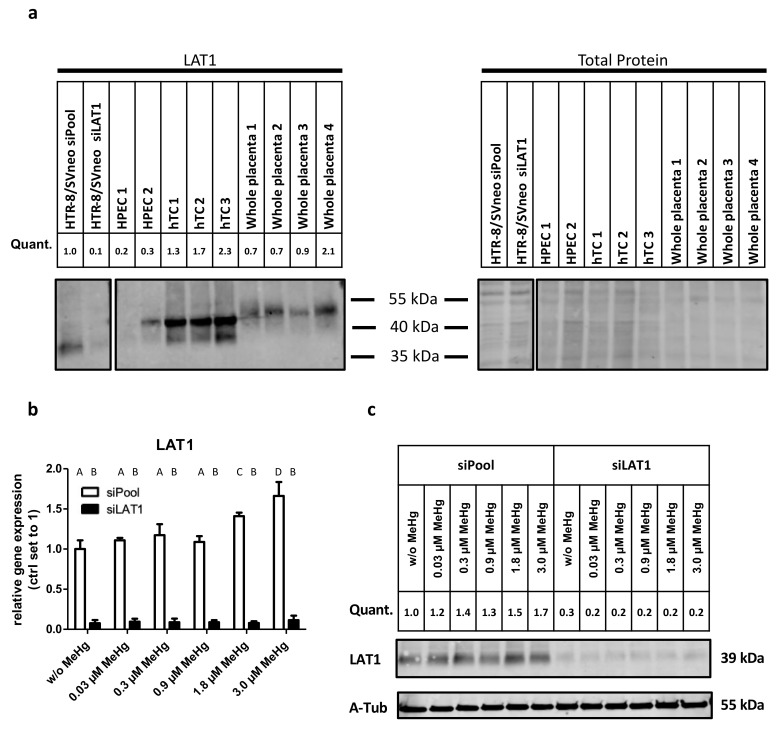
L-type amino acid transporter (LAT)1 protein expression in different types of placental cells and LAT1 knockdown verification in HTR-8/SVneo cells. (**a**) LAT1 protein expression in first trimester placental cell line HTR-8/SVneo in comparison to human primary placental endothelial cells (HPEC), human primary trophoblast cells (HPECs, hTCs), and whole placenta. LAT1 mRNA levels, analyzed by (**b**) qPCR and (**c**) protein levels determined by immunoblot in control (siPool) or siRNA-mediated LAT1-knockdown cells (siLAT1) treated with methylmercury (MeHg). LAT1 quantification (Quant.) was normalized to total protein (**a**) or alpha-tubulin (A-Tub) (**c**). Gene expression data represent mean values ± SD from three independent experiments, each performed in triplicate. Letters A–D denote homogeneous subgroups derived from one-way ANOVA and Student–Newman–Keuls (S–N–K) post hoc test (*p* < 0.05).

The protein levels of LAT1 differed between placental cell types. Human primary trophoblast cells (hTCs) showed higher LAT1 levels than primary endothelial cells (HPECs) (Figure 1a). HTR-8/SVneo cells expressed comparable LAT1 amounts as hTCs or whole placental tissue. The entire immunoblot is shown in Figure S1. The observed size discrepancies in the immunoblots for LAT1 between the cell types might be explained by different posttranslational modifications [25].

After siRNA-mediated gene knockdown in HTR-8/SVneo cells, LAT1 expression was determined by qPCR (Figure 1b) and immunoblot analysis (Figure 1c). LAT1 mRNA levels were reduced to 10–15% following transfection with LAT1-targeting siRNA in comparison to the nontargeting control (Figure 1b). Consistent with mRNA data, LAT1 protein levels were also reduced to 20% in LAT1-depleted cells (Figure 1c; the whole immunoblot is shown in Figure S2).

In HTR-8/SVneo control cells (without LAT1 knockdown), treatment with 1.8 µM and 3.0 µM MeHg raised the levels of LAT1 mRNA up to 60% at 3.0 µM MeHg (Figure 1b) and LAT1 protein up to 70% at 3.0 µM MeHg (Figure 1c). In order to address compensatory mechanisms of other system L subunits, we analyzed LAT2 and 4F2hc. Their expression mRNA levels were not affected by LAT1 depletion and/or the employed MeHg doses (Figure S3).

### 2.2. LAT1 Downregulation of MeHg-Treated HTR-8/SVneo Cells Decreases Cellular Hg Content and Cell Viability but Increases Cytotoxicity and Apoptosis

**Figure 2:**
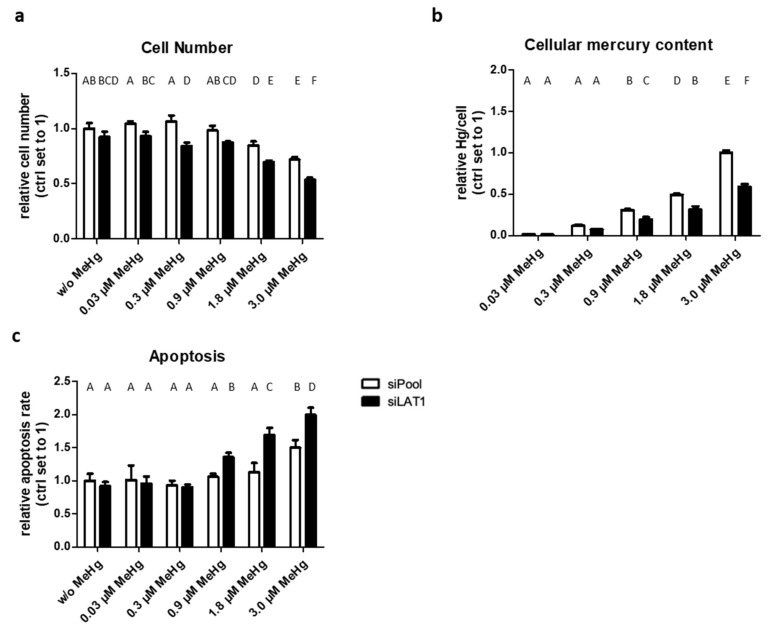
The effect of LAT1 knockdown on cell number, cellular Hg levels, and apoptosis rate in MeHg-exposed HTR8-SVneo cells. Cell number (**a**), cellular Hg content (**b**), as well as apoptosis rate (**c**) in control (siPool) and LAT1-depleted (siLAT1) cells. The data represent mean values ± SD from three independent experiments, each performed in triplicate. Letters A–F denote homogeneous subgroups derived from one-way ANOVA and S–N–K post hoc test (*p* < 0.05).

Treatment of HTR-8/SVneo cells with constitutive LAT1 expression with MeHg at concentrations ≥1.8 µM for 72 h led to a reduction in cell number to ~70% of untreated cells (Figure 2a). This effect was aggravated by LAT1 depletion, where already 0.3 µM MeHg reduced the cell number relative to the control group. MeHg-treated cells accumulated the metal in a dose-dependent manner (Figure 2b). LAT1 knockdown cells accumulated up to 40% less Hg (total Hg was analyzed) than control cells. In control cells, apoptosis was induced at 3.0 µM MeHg, whereas after LAT1, depletion increased apoptosis was present already at 0.9 µM MeHg (Figure 2c).

**Figure 3:**
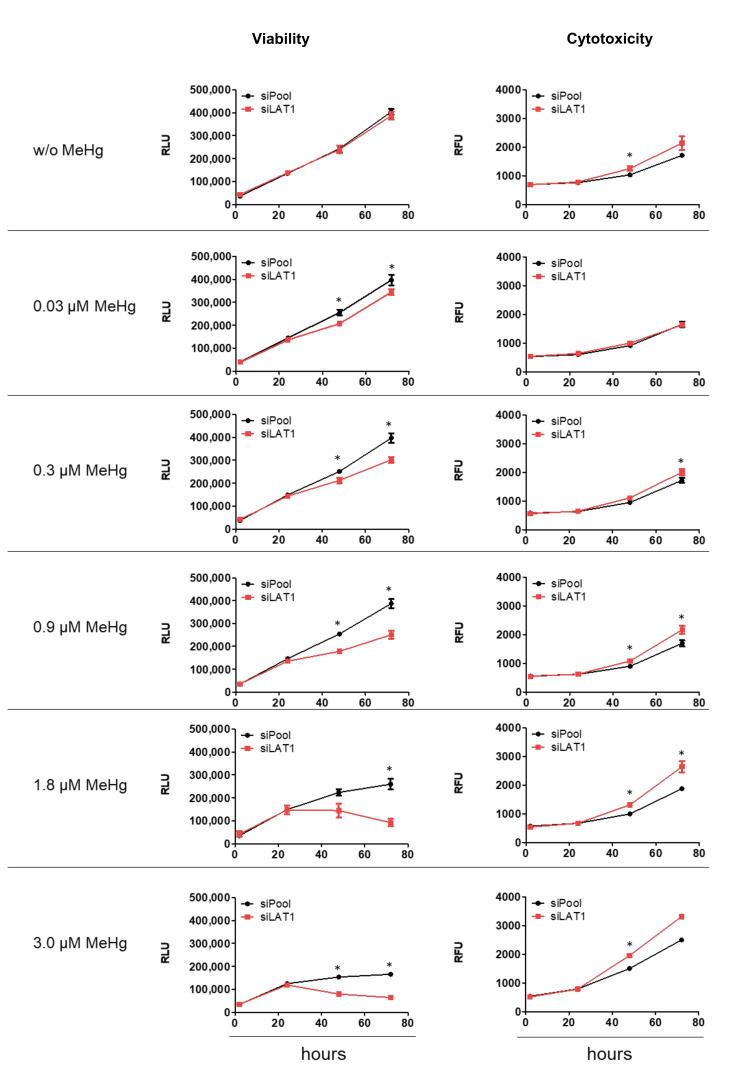
Cell viability and cytotoxicity in control (siPool) and LAT1-depleted (siLAT1) HTR8-SVneo cells after MeHg treatment for 72 h. RLU: Relative Luminescence Unit; RFU: Relative Fluorescence Unit. The data represent mean values ± SD from three independent experiments, each performed in triplicate. * *p* < 0.05 from Student’s *t*-test.

Without MeHg treatment, cell viability and cytotoxicity were not affected by LAT1 knockdown after 72 h, although a trend for increased cytotoxicity was observed (Figure 3, upper panel). High concentrations (≥1.8 µM) of MeHg decreased cell viability down to 50% of unexposed cells and increased cytotoxicity up to 125% of nontreated cells. In LAT1-depleted cells, however, cell viability began to decrease already at a dose ≥0.03 µM MeHg, and cytotoxicity was increased at a dose of ≥0.3 µM MeHg (Figure 3).

### 2.3. LAT1 Knockdown Decreases GSH Levels and Promotes Oxidative Stress

**Figure 4:**
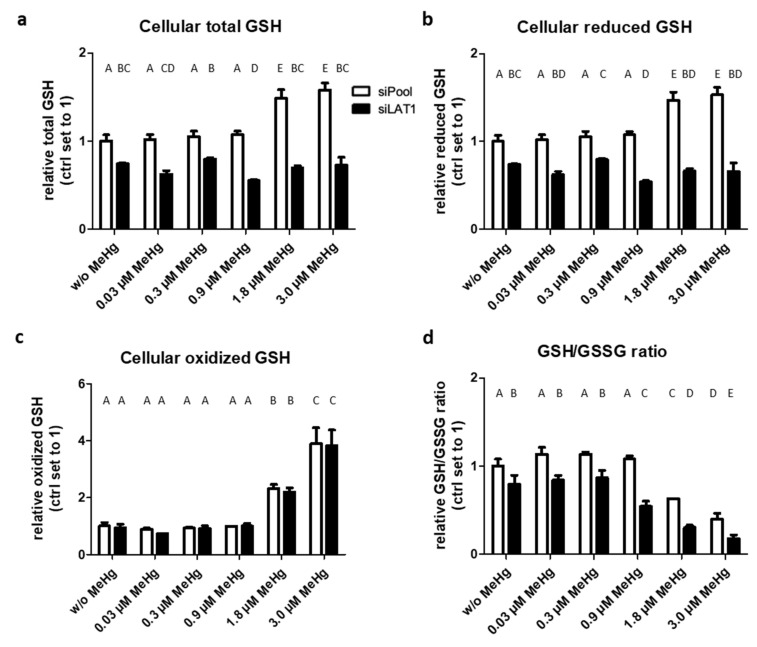
Effects of MeHg treatment and LAT1 downregulation on GSH status in HTR-8/SVneo cells. Exposure to MeHg led to dose-dependent increases in total glutathione (GSH) (**a**), reduced GSH (**b**), and oxidized GSH (GSSG) (**c**) and decreased the GSH/GSSG ratio (**d**) relative to controls (w/o MeHg). The LAT1 knockdown (siLAT1) reduced GSH levels, and thereby the GSH/GSSG ratio compared to control cells (siPool). The data represent mean values ± SD from three independent experiments, each performed in triplicate. Letters A–E denote homogeneous subgroups derived from one-way ANOVA and S–N–K post hoc test (*p* < 0.05).

In control cells with constitutive LAT1 expression, total GSH and reduced GSH levels were increased only at high MeHg doses (≥1.8 µM), reaching levels of ~160% relative to unexposed controls (Figure 4a,b). However, depletion of LAT1 decreased total and reduced GSH levels to ~70% of control cells relatively independently of MeHg treatment; even concentrations of ≥1.8 µM failed to increase them. Oxidized GSH levels also increased only at MeHg concentrations of ≥1.8 µM (up to 400% of the unexposed group after 3 µM MeHg treatment), but surprisingly, there was no difference between control and LAT1 knockdown cells regardless of MeHg treatment (Figure 4c). MeHg concentrations of ≥1.8 µM decreased the GSH/oxidized GSH (GSSG) ratio in control cells to ~40% of the unexposed group after 3 µM MeHg treatment. In LAT1 knockdown cells this effect was more pronounced and was, according to the findings described above, present also in cells that were not exposed to MeHg (Figure 4d). The extent of decrease in cellular MeHg uptake and GSH synthesis upon LAT1 knockdown was found to depend on LAT1 expression levels (Figure S4).

### 2.4. Cysteine and Methionine Availability Affect the Oxidative Stress Response

**Figure 5:**
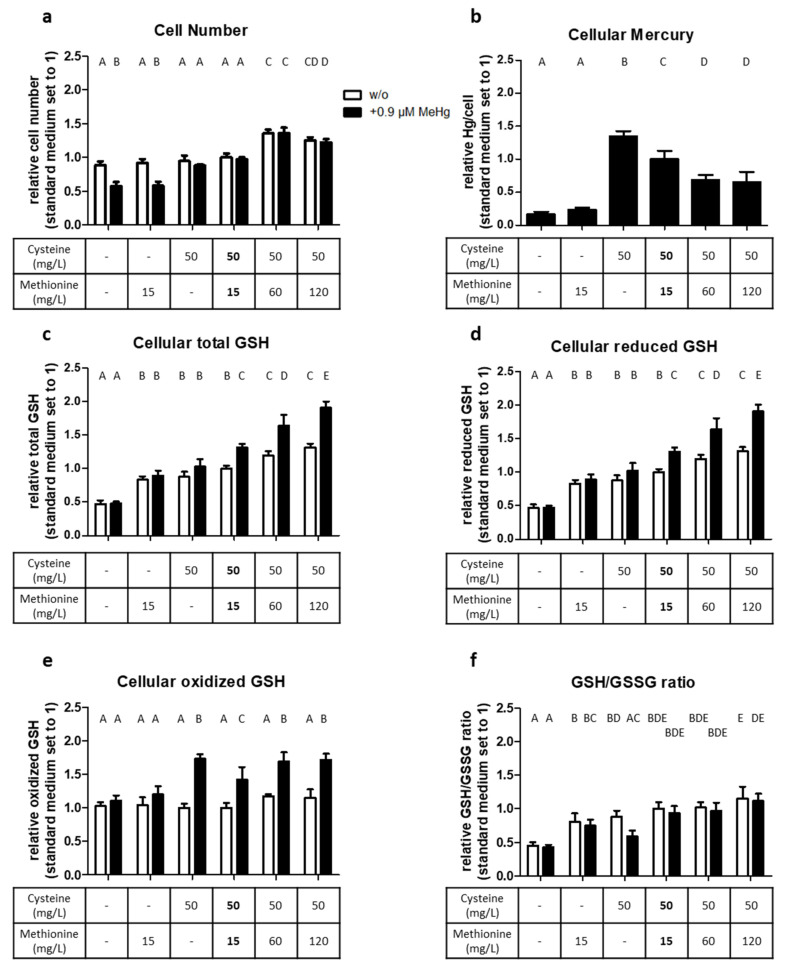
Effect of cysteine and methionine on cell number, Hg levels, and GSH status of HTR-8/SVneo cells. Cells were cultured for 24 h in media with different amino acid levels of cysteine and methionine (the standard medium containing 50 mg/L cysteine and 15 mg/L methionine are in bold numbers), either in the presence of 0.9 µM MeHg or without MeHg (w/o). In addition to cell number (**a**) and cellular Hg levels (**b**), cellular total GSH, reduced GSH, oxidized and GSH/GSSG ratio (**c–f**) were analyzed as indicators for oxidative stress. The data represent mean values ± SD from three independent experiments, each performed in triplicate. Letters A–E denote homogeneous subgroups derived from one-way ANOVA and S–N–K post hoc test (*p* < 0.05).

There were two notable effects of cysteine/methionine supply on the number of HTR-8/SVneo cells. First, cell number increased sharply when more methionine (60 or 120 mg/L) was offered than present in standard medium (15 mg/L methionine; ~100 µM). This was the case at constant cysteine supply (50 mg/L; ~208 µM) and independent of MeHg treatment. Second, exposure to 0.9 µM MeHg reduced cell number only when cysteine was absent from the medium. This was independent of whether methionine was present or not (Figure 5a).

The cysteine/methionine availability also affected cellular Hg concentrations. The cells accumulated significantly less Hg when cultured in the absence of cysteine. Concomitant methionine depletion had no further effect. However, when cells were kept under cysteine-replete but methionine-deplete conditions, cellular Hg levels even raised to ~140%. In contrast, cellular Hg levels were reduced to ~60% of cells kept in standard medium during cysteine-replete and methionine-enriched culture conditions (Figure 5b).

Next, we wanted to determine the impact of cysteine and/or methionine depletion on oxidative stress levels. Different cysteine/methionine supplies had the same effects on levels of total GSH (Figure 5c) and reduced GSH (Figure 5d); namely, a significant increase in GSH levels only when both amino acids were supplied (corresponding to a reduction when neither amino acid was present) and when treated with MeHg. It is noteworthy that the levels of oxidized GSH (Figure 5e) increased only in the presence of MeHg whenever cysteine was present. A lowered GSH/GSSG ratio to ~40% of cells cultured in standard medium was present when both cysteine and methionine were absent; this was independent of MeHg exposure (Figure 5f). MeHg treatment led to a lower GSH/GSSG ratio particularly in methionine-deplete condition.

## 3. Discussion

### 3.1. LAT1 Is Required for MeHg Uptake but also for Protection from the Metal’s Toxicity

Although several proteins could be involved in cellular MeHg uptake, in the present work we focused on LAT1 because the transporter had the strongest effect on cellular Hg levels in a previous work on trophoblast cell culture models [4]. The special importance of LAT1 for MeHg uptake is further demonstrated in *Xenopus* oocytes transfected with LAT1 that showed higher uptake levels of the metal than those expressing LAT2 [5]. As expected, we found reduced cellular Hg levels in LAT1-depleted HTR-8/SVneo cells. To our surprise, these cells accumulated less MeHg but still showed reduced cell number and viability. On the other hand, cytotoxicity and oxidative stress were highly increased. This suggests that despite their lower intracellular MeHg levels, the cells are less capable to handle the metal’s toxicity. In general, the susceptibility to MeHg toxicity strongly differs between cell types (Table 1).

It has to be noted that MeHg concentrations used in in vitro studies (up to 15 µM) are far higher than those present in pregnant women, where they range from 0.22 µg/L (~1 nM) to 38.1 µg/L (~177 nM) [30]. However, chronic MeHg exposure occurs in vivo over a period of nine months, and the metal accumulates in the placenta [31], which could generate levels able to harm placental cells.

One reason for the pronounced sensitivity to MeHg toxicity in LAT1-depleted HTR-8/SVneo cells may be an impaired antioxidative response as a result of reduced GSH levels. GSH is a tripeptide made up by glutamate, cysteine, and glycine, and especially cysteine availability is considered a rate-limiting factor in GSH synthesis [32]. The main route of cysteine uptake occurs via the import of extracellular cystine (oxidized dimer of cysteine) at the expense of one glutamate molecule by the Xc− system, composed of xCT transporter and 4F2hc. Imported cystine is then reduced to cysteine by cystine reductase [24]. However, there are other possibilities for how the cell could acquire cysteine via LAT1. First, LAT1 is involved in the uptake of many essential amino acids, including methionine [33]. Methionine can then be converted to cysteine via the transsulfuration pathway [24]. Second, after its import, MeHg dissociates from L-cysteine. This cysteine could now be available for biosynthetic pathways. This might result in diminished cellular cysteine levels in LAT1-deficient cells, which would explain reduced GSH synthesis and higher vulnerability to oxidative stress. In line with this hypothesis, MeHg concentrations ≥1.8 µM elevated oxidative stress and increased LAT1 expression as well as GSH synthesis in HTR-8/SVneo cells. A similar induction of LAT1 expression by oxidative stressors has been described in H_2_O_2_-treated human cholangiocarcinoma cells [34] and MeHg-exposed mouse muscle cells [35].

Our results clearly show a negative additive effect of MeHg exposure and LAT1 deficiency. This can be interpreted in a way that genetic predisposition affecting proper LAT1 expression and function may increase vulnerability to additional (oxidative) stress. We want to emphasize that these effects occurred at physiological concentrations (0.03 µM MeHg) within a relatively short period of time, i.e., 24 h.

The global importance of LAT1 is evident in LAT1 knockout mice that were embryonically lethal in the homozygous but not in the heterozygous state [36]. Furthermore, inhibition of LAT1 reduced cell viability in human umbilical vein endothelial cells, human primary aortic smooth muscle cells, and two human renal cancer cell lines [37,38]. While LAT1 deficiency is involved in many pathophysiological disorders, it may also be relevant for pregnancy conditions, such as fetal growth restriction (FGR) [39,40]. However, a recent study found elevated LAT1 levels in FGR and preeclamptic placentas, which the authors interpreted as a compensatory mechanism [41]. This is in line with our results, thus strengthening the notion of a regulating role of LAT1 in these conditions by supplying amino acids for GSH synthesis to ameliorate accompanying oxidative stress [42,43].

This essential role of LAT1 in the human placenta is further demonstrated by its high expression in the organ [44], where it mainly localizes in the STB [4]. We have also shown that hTCs show higher LAT1 protein levels than HPECs, suggesting that LAT1 may be of greater importance for amino acid transport into STB than into fetal endothelial cells. In line with this, a role for LAT1 in proper placentation [45] and STB development [46] has been previously demonstrated.

### 3.2. Cysteine and Methionine Are Required for GSH Synthesis

As expected, cells cultured in cysteine-depleted medium accumulated less MeHg since the metal cannot form MeHg-L-cysteine complexes required for its uptake [3]. Similar to LAT1 deficiency, cells cultured in the absence of cysteine accumulated less MeHg but were highly vulnerable to MeHg toxicity, as indicated by their reduced cell number. A reason may be that without cysteine the cells cannot produce adequate GSH levels to cope with MeHg. However, we found that GSH synthesis and GSH/GSSG ratio were only reduced in absence of cysteine and methionine regardless of MeHg treatment. This suggests that as long as either methionine or cysteine are present the cell can synthesize adequate amounts of GSH [47,48]. Surprisingly, cysteine-depleted cells were reduced in cell numbers even though their GSH status suggested sufficient capacity for defense against oxidative stress. However, a recent model suggested that cysteine depletion may lead to GSH-independent ferroptotic cell death, which might explain why these cells are still highly vulnerable to oxidative stress [24].

Interestingly, intracellular Hg levels increased more in the presence of cysteine and in the absence of methionine than when both amino acids were offered. This effect can be explained in two ways. First, the absence of methionine in the medium increased both mRNA and protein expression of LAT1 (Figure S5) to increase absorption of methionine. So far, LAT1 upregulation through amino acid starvation has only been described in leucine-depleted mouse trophoblast stem cells [49]. However, with increased LAT1 expression, MeHg-L-cysteine uptake also increases. Second, MeHg-L-cysteine does not have to compete with methionine as a LAT1 substrate (MeHg-L-cysteine has higher affinity to LAT1 than methionine) and can therefore be taken up even more efficiently [5,50]. Accordingly, an increase in methionine levels reduced the cellular uptake of MeHg into HTR-8/SVneo cells. Based on these results we speculate that supplementation with methionine could protect from MeHg toxicity [50,51], as it reduces the uptake of MeHg-L-cysteine complexes and improves the oxidative stress response by enabling GSH synthesis.

## 4. Material and Methods

### 4.1. Cell Culture

HTR-8/SVneo cells (ATCC, CRL-3271™, Lot# 64275781, Manassas, VA, USA) were cultivated in RPMI-1640 medium (Gibco; 31870074, Carlsbad, CA, USA) containing 5% fetal bovine serum (FBS; PanBiotech; P40-38100, Aidenbach, Germany) and 1% glutamax (Gibco; 35050061, Carlsbad, CA, USA). The cells were sub-cultured every 3–5 days. CASY cell counter and analyzer (CASY; Innovatis Technologies Inc., Fairfax, VA, USA) was used to determine the cell number. The conditions for cell cultivation in the incubator were 37 °C and 5% CO_2_. Mycoplasma contamination was tested on a regular basis (MycoAlert; Lonza, Basel, Switzerland). HTR-8/SVneo cells from passages 86 to 96 were used.

Human placental endothelial cells (HPEC) and human trophoblast cells (hTC) used in the immunoblot (Figure 1) were isolated from healthy placentas according to previous studies [4,52].

### 4.2. Methylmercury (MeHg) Dosages

MeHg dissolved in water (Alfa Aesar; 33,553.AC, Haverhill, MA, USA) was used in the experiments. The concentrations ranged from a noncytotoxic dose (0.03 µM; corresponds to about 6 µg/L) to a highly cytotoxic dose (3 µM; corresponds to 645 µg/L) [4,53]. Treatment with 0.9 µM (194 µg/L) MeHg was used as a standard concentration in later experiments, as this dosage had no cytotoxic effects and showed good detectability of cellular Hg levels after 24 h exposure.

### 4.3. siRNA Mediated Knockdown

Cells were seeded in 6-well plates at a density of 1 × 10^5^ cells/well. On the next day, cells were transiently transfected with nontargeting siRNA (siPool = controls; GE Dharmacon; D-001810-10-20, Lafayette, IN, USA) and LAT1/SLC7A5-specific siRNA (GE Dharmacon; L-004953-01-0020, Lafayette, IN, USA) using Lipofectamine RNAiMAX (Life Technologies; 13778075, Carlsbad, CA, USA), as described in previous studies [19,54]. siRNA concentration of 50 nM was used in all experiments. In an additional experiment, various siRNA concentrations were tested in combination with treatment of cells with 0.9 µM MeHg (see Figure S4). Cells were cultured for 58 h and after this time MeHg (0.03 µM, 0.3 µM, 0.9 µM, 1.8 µM, 3 µM) was added for 72 h.

### 4.4. Analysis of Total Hg

Cells and reference material (trace elements urine L-2, solder 1403081) were digested with nitric acid (69%; Suprapur; Roth; HN50.3, Karlsruhe, Germany) in a microwave oven (MARS6, CEM Corporation; Matthews, NC, USA) and analyzed for total MeHg by atomic fluorescence spectroscopy (AFS; mercur plus, Analytic Jena, Jena, Germany). The concentrations of the reference material (46.44 ± 0.53 µg/L; *n* = 2; recovery 105 ± 1%) were within the certified range (Hg: 44.0 µg/L, range: 35.2–52.9 µg/L). The detection limit was 0.016 µg/L (*n* = 3). All samples were measured in duplicate (RSD < 1%) at the appropriate dilution and the concentrations were calculated from a standard curve (0.0–3.2 µg/L MeHg).

### 4.5. Cytotoxicity and Cell Viability Assays

Cell viability and cytotoxicity were measured simultaneously in a 96-well plate. RealTime-Glo MT Cell Viability Assay (Promega; G9711, Madison, WI, USA) was used to determine the cell viability, while cytotoxicity was measured by CellTox Green Cytotoxicity Assay (Promega; G874, Madison, WI, USA) according to previous studies [21]. In brief, 1 × 10^3^ cells/well were seeded, treated with MeHg (0.03 µM, 0.3 µM, 0.9 µM, 1.8 µM, 3 µM) the following day, and analyzed after 2 h, 24 h, 48 h and 72 h after treatment. The performance of the assay, i.e., reduction of cell viability and increase of cytotoxicity, was controlled with 10 µM ionomycin (Sigma; I9657-1MG, St. Louis, MO, USA) according to the manufacturer’s protocol (Figure S6a,b).

### 4.6. Apoptosis Assay

Caspase-Glo 3/7 Assay System (Promega; G8090, Madison, WI, USA) was used to determine apoptosis in 96-well plates according to the manufacturer’s instructions. In brief, 1 × 10^3^ cells/well were seeded, on the following day treated with MeHg (0.03 µM, 0.3 µM, 0.9 µM, 1.8 µM, 3.0 µM), and caspase activity was measured 48 h posttreatment. Staurosporine (1 µM; Sigma; S5921-.5MG, St. Louis, MO, USA) was used as positive control (Figure S6c).

### 4.7. GSH/GSSG Assay

As the ratio between reduced and oxidized forms of GSH is an important and dynamic indicator of oxidative stress and redox environment [55,56], the GSH/GSSG-Glo Assay (Promega; V6611, Madison, WI, USA), a luminescence-based system that quantifies total glutathione (GSH) and oxidized GSH (GSSG) in cultured cells, was used to determine oxidative stress of MeHg-treated HTR-8/SVneo cells. Reduced GSH was calculated according to the manufacturer’s protocol (total GSH—oxidized GSH (GSSG) = reduced GSH). GSH measurement was normalized to cell number.

### 4.8. RNA Isolation, cDNA Synthesis and Quantitative PCR

TRI reagent (Sigma; 93289-100ML, St. Louis, MO, USA) was used to isolate RNA according to the manufacturer’s instructions. Total RNA was reverse transcribed using Go-Script Reverse Transcription System (Promega; A5001, Madison, WI, USA). cDNA was diluted 1:10 and 2 µL cDNA solution was used as template in gene expression assay reactions, following Applied Biosystems StepOnePlus Real-Time PCR System protocol. The employed primers were Hs01001189_m1 (SLC7A5), Hs00824723_m1 (UBC), Hs00374243_m1 (SLC3A5), and Hs00794796_m1 (SLC7A8) (Thermofisher, Waltham, MA, USA).

### 4.9. Immunoblotting

Cells were lysed in RIPA Buffer (Thermo Scientific; 89901, Waltham, MA, USA) supplemented with Halt Protease and Phosphatase Inhibitor Cocktail (Thermo Scientific; 78420) and 0.5 M EDTA solution (Thermo Scientific; 78430). Protein concentrations were determined using Bradford reagent (BioRad; 500006, Hercules, CA, USA). Total protein (17.5 µg) was separated by 12.0% SDS-PAGE and transferred onto Odyssey Nitrocellulose Membranes (LI-COR, 926-31090, Lincoln, NE, USA). Membranes were dried for 10 min at 37 °C and then blocked for 1 h in Odyssey Blocking Buffer (tris-buffered saline: TBS) (LI-COR, 927-50000, Lincoln, NE, USA). Blots were incubated in TBS containing 0.1% Tween-20 (TBST) and the alpha tubulin (Merck; CP06; 1:1000; anti-mouse, Darmstadt, Germany) and/or LAT1 (Cell Signaling; 5357S; 1:1000; anti-rabbit, Danvers, MA, USA) primary antibody overnight at 4 °C. On the next day, blots were washed with TBST and incubated with the secondary fluorophore-conjugated antibody (LI-COR; anti-mouse IR-Dye680; #92568070; 1:20,000/anti-rabbit IR-Dye800; 92632211; 1:20,000) for 1 h at room temperature. The secondary antibodies were detected with the Odyssey CLx imager (LI-COR, Lincoln, NE, USA) using Image Studio Lite 5.2 software. REVERT 700 Total Protein Stain (LI-COR; 926-11010, Lincoln, NE, USA) was used to detect total protein. 

### 4.10. Statistics

Data were obtained from at least three independent experiments (three passages) made in triplicate and represent mean values ± standard deviation (SD). One-way ANOVA was used for statistical analysis of group differences, followed by a Student–Newman–Keuls (S–N–K) test to correct for multiple testing (homogeneous subgroups are labelled with the same letters in the graphs). Parametric Student´s *t*-test was applied for statistical analysis of cell viability and cytotoxicity. Statistical calculations were performed by using IBM SPSS26, and graphs were created in GraphPad Prism 6 software. The significance level was set to α = 0.05.

## 5. Conclusions

In the present study we show for the first time that LAT1 is required for protection against MeHg toxicity in HTR-8/SVneo cells. Although LAT1-deficient cells accumulate less MeHg, they are impaired in their GSH synthesis. Consequently, the cells are highly susceptible to oxidative stress even at physiological MeHg concentrations. Increased MeHg exposure in combination with (genetically) reduced placental LAT1 function could seriously impair essential placental functions.

## Figures and Tables

**Table 1:** Reduction of cell number after MeHg treatment in different cell lines.

Cell Type	Species	MeHg (µM)	Exposure Time	Cell Number Reduction	Reference
HTR-8/SVneo	human	3.0	72 h	~40%	[21]
HTR-8/SVneo	human	4.6	72 h	~50%	[23]
HTR-8/SVneo	human	7.8	24 h	~30%	[23]
BeWo cells	human	3.0	24 h	~30%	[4]
Cerebellar granule neurons	rat	1.9	24 h	~50%	[26]
SH-SY5Y	human	2.8	24 h	~50%	[26]
Endothelial cells	human	4.1	24 h	~50%	[27]
Glioma cells	rat	6.1	24 h	~40%	[28]
P12 cells	rat	14.3	24 h	~40%	[28]
Astrocytes	human	15.0	24 h	~30%	[29]

## Data Availability

The manuscript, together with Supplementary Materials, contains all data supporting the results of this study.

## Supplementary Materials

The following are available online at https://www.mdpi.com/1422-0067/22/4/1707/s1.
