# Supplementary material for: Amino Acid Transporter LAT1 (SLC7A5) Mediates MeHg-Induced Oxidative Stress Defense in the Human Placental Cell Line HTR-8/SVneo
Source: Int J Mol Sci. 2021 Feb 8;22(4):1707. doi: 10.3390/ijms22041707 (PMC7915079; doi:10.3390/ijms22041707)
Supplement: Supplementary file 1 [file ijms-22-01707-s001.pdf]

International Journal of
Molecular Sciences

Supplementary Material

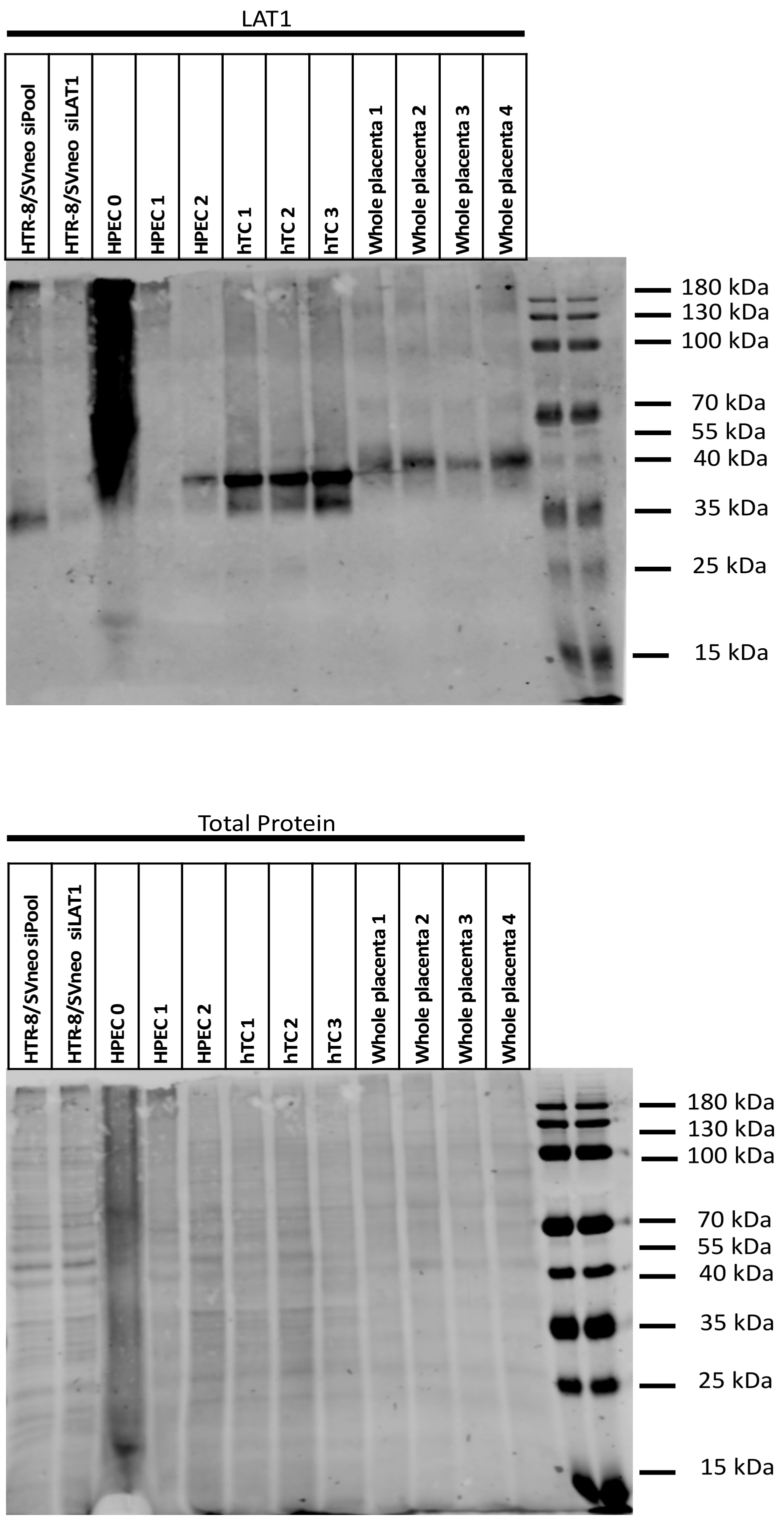

Figure S1. Total immunoblot of LAT1 protein in placental cells and whole placenta lysates.

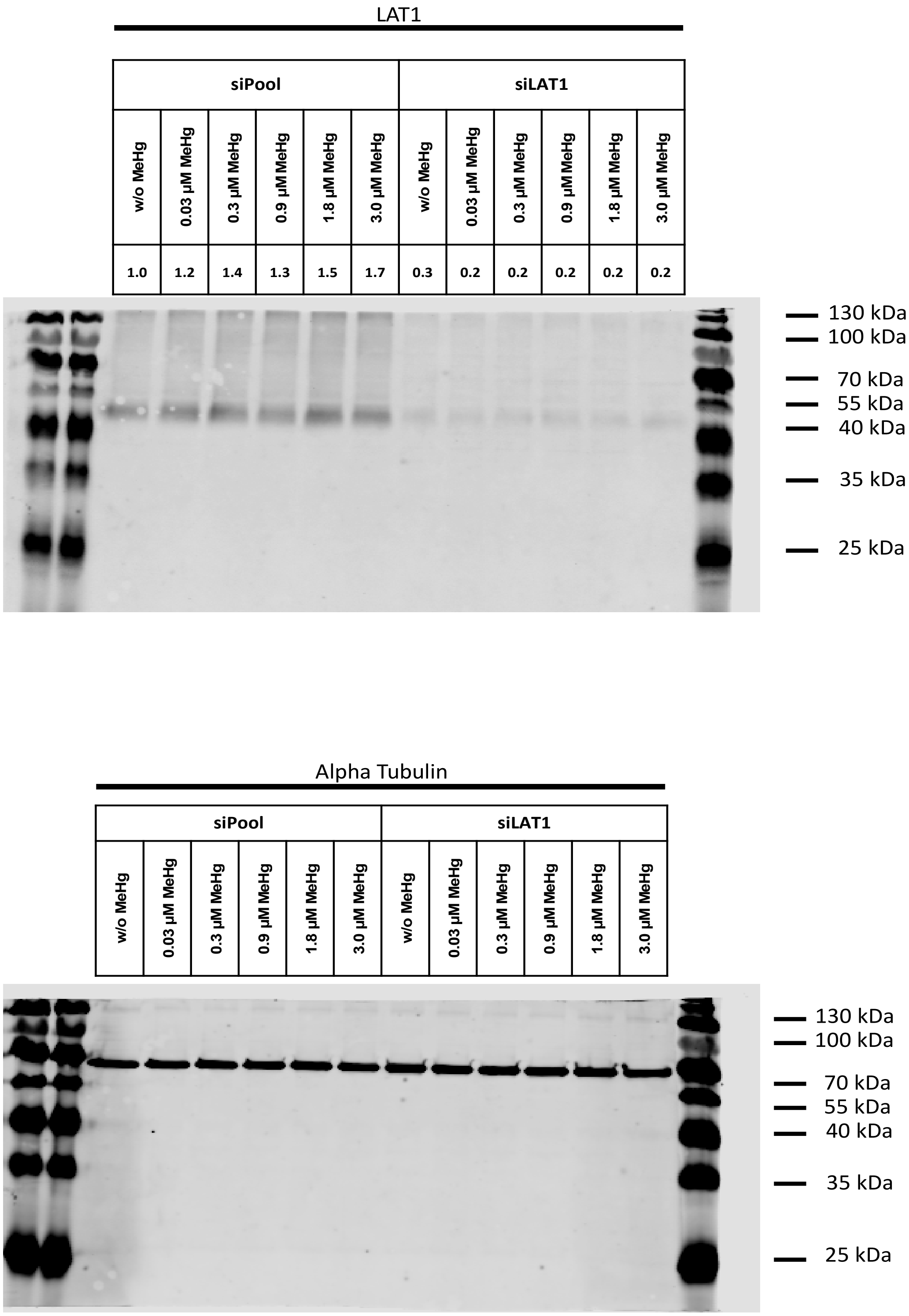

Figure S2. Total immunoblot to verify efficient siRNA-mediated gene knockdown of LAT1 compared to control cell.

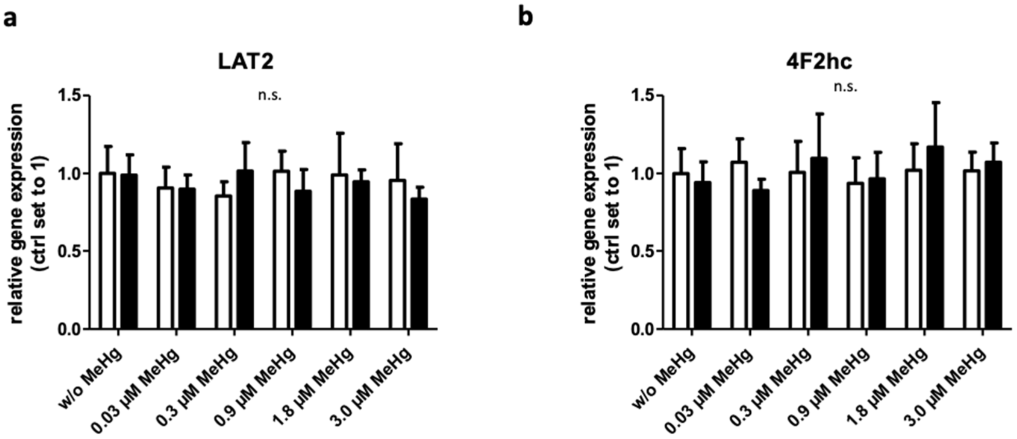

Figure S3. MeHg exposure does not affect mRNA expression of **(a)** LAT2 (SLC7A8) and **(b)** 4F2hc (SLC3A2). The data represent mean values ± SD from three independent experiments, each performed in triplicate. White bars: siPool; Black bars: siLAT1. Statistical analyses used one-way ANOVA and S-N-K posthoc test ($p < 0.05$). $n = 3$; n.s.= non-significant.

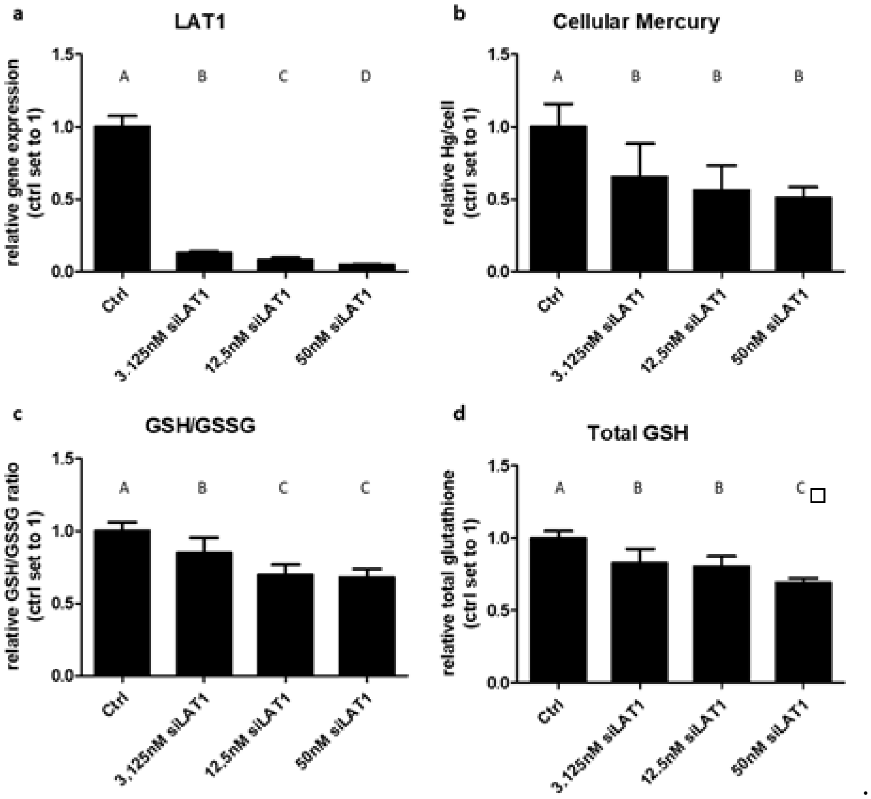

Figure S4. The amount of LAT1-specific siRNA affects LAT1 mRNA levels **(a)**, cellular Hg content **(b)**, GSH/GSSG ratio **(c)** as well as Total GSH **(d)**. The data represent mean values ± SD from three independent experiments, each performed in triplicate. Letters A–D denote homogeneous sub-groups derived from one-way ANOVA and S-N-K posthoc test ($p < 0.05$). $n = 3$.

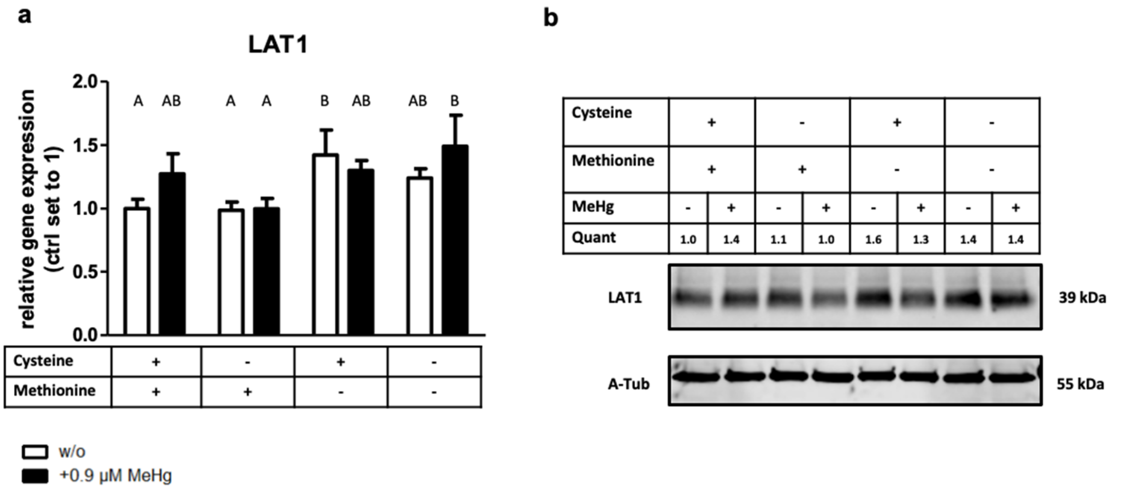

Figure S5. LAT1 mRNA **(a)** and protein levels **(b)** were determined in HTR-8/SVneo cells cultured for 24h in media with different levels of cysteine (50 mg/L) and methionine (15 mg/L), either in the presence of 0.9 µM MeHg or without MeHg (*w/o*). Letters A,B denote homogeneous subgroups derived from one-way ANOVA and S-N-K posthoc test ($p < 0.05$). n = 3; LAT1 protein expression was normalized to α-tubulin (A-tub) levels (representative immunoblot shown).

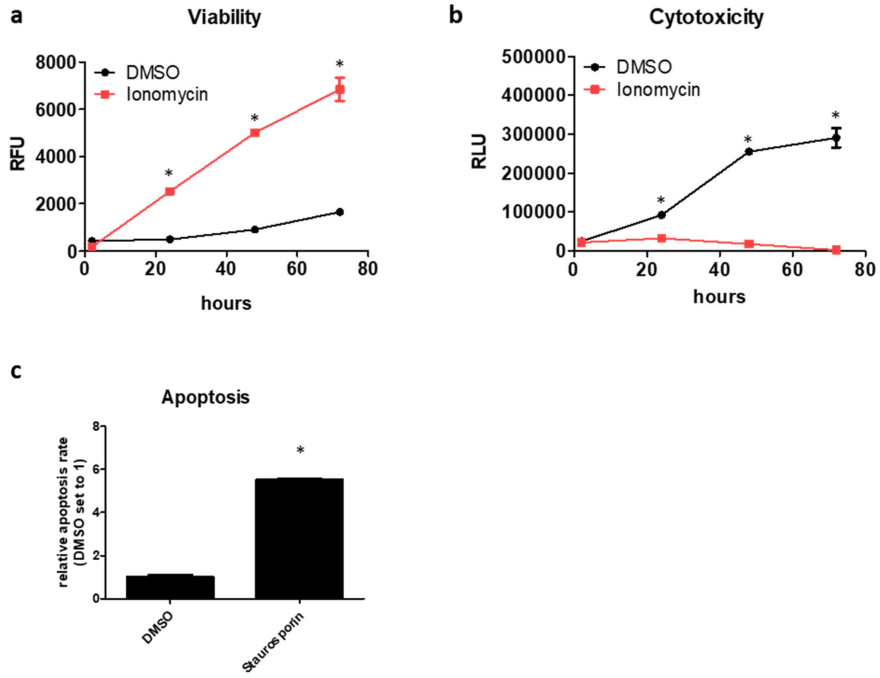

Figure S6. Positive controls of viability (**a**), cytotoxicity (**b**) and apoptosis (**c**). The data represent mean values ± SD from three independent experiments, each performed in triplicate. *$p < 0.05$ from Student's *t*-test. 1% Dimethylsulfoxid (DMSO) in medium was used as control.
